# Supplementary material for: A core outcome set for research and clinical practice in women with pelvic girdle pain: PGP-COS
Source: PLoS One. 2021 Feb 25;16(2):e0247466. doi: 10.1371/journal.pone.0247466 (PMC7906405; doi:10.1371/journal.pone.0247466)
Supplement: S1 Table — (DOCX) [file pone.0247466.s001.docx]

**S1 Table. List of potential outcome measures including additional outcomes identified in Round 1 of Delphi Study**

|  | **Outcome Measure** | **Definition and/or example** |
| --- | --- | --- |
| *Life Impact Outcomes* | Pain behaviour | actions that indicate someone is experiencing pain for example grimacing, verbal confirmation, moaning, holding site of pain, etc. |
|  | Pain character/type | for example, aching, stabbing, etc. |
|  | Pain frequency | - |
|  | Pain intensity/severity | - |
|  | Pain location | - |
|  | Full pain recovery | being pain free/no longer in pain after treatment |
|  | Function/disability/activity limitation | including sitting, standing, walking, stairs, carrying things, etc. |
|  | Physical activity levels/ exercise limitations | - |
|  | Need for mobility aid | for example, crutches or wheelchair, etc. |
|  | Perceived body imbalance | for example, feeling that body is twisted, one leg is shorter, body “feels” off, etc. |
|  | Sexual functioning | impact of PGP on person’s ability to engage in sexual/intimate activity physically/mentally |
|  | Health related quality of life | a person’s perception on how PGP impacts her physical/psychological/social health |
|  | Health status | measure of a person’s physical/psychological/social aspects of health |
|  | Family life impact | including relationship to partner, family and/or other children and the ability to care for other children |
|  | Social life impact | person’s ability to interact with others and participate in desired social life activities (for example, going out with friends) |
|  | Patient satisfaction with life | - |
|  | Patient satisfaction with treatment | - |
|  | Patient expectation of treatment | - |
|  | Anxiety | excessive worry and/or fear |
|  | Confidence | - |
|  | Depression | feelings of sadness, loss of interest or enjoyment |
|  | Dependence on others | during activities |
|  | Emotional symptoms | including mood and emotional distress |
|  | Fear avoidance | fear of movement resulting in avoidance of movement |
|  | Frustration | - |
|  | **Outcome Measure** | **Definition and/or example** |
|  | Pain catastrophizing | exaggerated and/or repeated negative thoughts about their pain and the future of it |
|  | Self-efficacy | a person’s belief about their ability to perform certain tasks/activities |
|  | Well-being | a person’s positive perception of all aspects of life including happiness, contentment, etc. |
|  | Fatigue | tiredness |
|  | Sleep function | including sleep disturbance, trouble sleeping, painful sleeping positions, poor sleep quality, etc. |
| *Economic Impact & Resource-Use Impact Outcomes* | Work ability | including absence of work, sick leave, temporary incapacity, etc. |
|  | Work performance | a measure of how well you can perform your required work/tasks/activities |
|  | Analgesia use | prescription/over the counter medication use for pain |
|  | Cost | direct/indirect costs of seeking healthcare |
|  | Healthcare utilisation | the extent/frequency of accessing healthcare services for example, visiting clinicians, imaging, further tests, including other therapies, etc. |
| *Pathophysiological Manifestations &*  *Clinical Test Outcomes* | Anthropomorphic outcomes | measurements of size and proportion of the human body for example, height, weight, BMI, etc. |
|  | Body flexibility | - |
|  | Functional mobility | for example, functional load transfer, Timed Up and Go score, etc. |
|  | Gait endurance | how long you can walk |
|  | Gait speed | how fast you can walk |
|  | New born outcomes | for example, Apgar score, birth weight, gestational week of delivery, etc. |
|  | Outcomes from pain provocation/location tests | - |
|  | Posture | - |
|  | Pubis Symphysis mobility | as measured through medical imaging |
|  | Maternal pregnancy outcomes | for example, gestation week at delivery, length of delivery, mode of delivery, etc. |
|  | Muscle endurance | how quickly a muscle becomes fatigued |
|  | Muscle strength | - |
|  | Recovery of symptoms | - |
|  | Step length | - |
|  | Surgical outcomes | for example, fluoroscopy time, insertion time for guide wires, operation time and screw position |
|  | Urinary Incontinence | leaking urine when don’t want to |
|  | **Outcome Measure** | **Definition and/or example** |
| *Adverse Events Outcomes* | Maternal adverse events/ undesirable side effects | - |
|  | Unborn/born child adverse events/undesirable side effects | - |
| *Additional Outcomes* | Breathing function | - |
|  | Clinical findings on motion palpation/ joint play of pelvic girdle joints | - |
|  | Coping strategies/coping styles | - |
|  | Global perceived improvements/global rate of change | measurement of self-perceived change in health status |
|  | Goal attainment | method of setting individual goals for a patient and then scoring the extent to which they are achieved in a course of an intervention |
|  | Likelihood of planning subsequent pregnancies due to risk of reoccurrence | - |
|  | Motor control/movement strategies/movement patterns | Including gait, muscle activation patterns, etc. |
|  | Muscle tightness |  |
|  | Need for additional supports | for example, back supports for sitting, mattress for sleeping, etc, |
|  | Outcomes from functional tests | for example, Active Straight Leg Raise, etc. |
|  | Pain duration/pain pattern | - |
|  | Patients beliefs about pain/meaning of complaints to patient | - |
|  | Patient understanding/ knowledge of PGP | - |
|  | Postural observation | for example, symmetry, dysfunction, etc. |
|  | Symptoms during menstruation | - |
