# Supplementary material for: A core outcome set for research and clinical practice in women with pelvic girdle pain: PGP-COS
Source: PLoS One. 2021 Feb 25;16(2):e0247466. doi: 10.1371/journal.pone.0247466 (PMC7906405; doi:10.1371/journal.pone.0247466)
Supplement: S2 Table — details the % of stakeholder group that rated an outcome as “important” (4+ on the 5-point rating scale survey or 7+ on the 9-point rating scale survey) above in each Delphi round. ± Outcome in final PGP-COS * Group 1 = clinician; 2 = clinician/researcher; 3 = patient; 4 = researcher; 5 = Service provider/policy maker a 5PT = participants responded to Delphi surveys using a 5-point rating Scale b 9PT = participants responded to Delphi surveys using a 9-point rating Scale. (DOCX) [file pone.0247466.s002.docx]

**S2 Table. Delphi Results**

|  |  | **Round 1** | | **Round 2** | | **Round 3** | |  |  | **Round 1** | | **Round 2** | | **Round 3** | |
| --- | --- | --- | --- | --- | --- | --- | --- | --- | --- | --- | --- | --- | --- | --- | --- |
| **Outcome**^±^ | **Group*** | 5PT^a^ | 9PT^b^ | 5PT^a^ | 9PT^b^ | 5PT^a^ | 9PT^b^ | **Outcome**^±^ | **Group*** | 5PT^a^ | 9PT^b^ | 5PT^a^ | 9PT^b^ | 5PT^a^ | 9PT^b^ |
| Pain behaviour | 1 | 74 | 50 | 65 | 55 | - | - | Patient expectation with treatment | 1 | 84 | 83 | 90 | 86 | - | 80 |
|  | 2 | 74 | 47 | 68 | 57 | - | - |  | 2 | 78 | 53 | 68 | 57 | - | 64 |
|  | 3 | 75 | 55 | 100 | 58 | - | - |  | 3 | 85 | 82 | 79 | 83 | - | 50 |
|  | 4 | 50 | 23 | 40 | 18 | - | - |  | 4 | 50 | 77 | 60 | 82 | - | 82 |
|  | 5 | 100 | 67 | 100 | 80 | - | - |  | 5 | 100 | 83 | 67 | 80 | - | 100 |
| Pain character/ type | 1 | 77 | 67 | 81 | 83 | 82 | 72 | Anxiety | 1 | 91 | 85 | 87 | 90 | 96 | 88 |
|  | 2 | 61 | 40 | 68 | 43 | 42 | 29 |  | 2 | 83 | 67 | 79 | 64 | 84 | 71 |
|  | 3 | 90 | 73 | 86 | 83 | 70 | 63 |  | 3 | 85 | 77 | 86 | 75 | 90 | 50 |
|  | 4 | 60 | 54 | 50 | 36 | 30 | 27 |  | 4 | 80 | 85 | 80 | 73 | 80 | 73 |
|  | 5 | 100 | 67 | 100 | 80 | 67 | 80 |  | 5 | 60 | 67 | 100 | 100 | 0 | 100 |
| **Pain frequency** | 1 | 95 | 83 | 94 | 97 | 96 | 84 | Confidence | 1 | 70 | 79 | 52 | 79 | - | - |
|  | 2 | 96 | 73 | 95 | 86 | 89 | 64 |  | 2 | 61 | 73 | 21 | 57 | - | - |
|  | 3 | 85 | 91 | 93 | 92 | 80 | 88 |  | 3 | 75 | 73 | 79 | 75 | - | - |
|  | 4 | 100 | 62 | 100 | 64 | 100 | 55 |  | 4 | 50 | 69 | 50 | 55 | - | - |
|  | 5 | 100 | 83 | 67 | 100 | 100 | 80 |  | 5 | 80 | 67 | 67 | 60 | - | - |
| **Pain intensity/ severity** | 1 | 93 | 81 | 100 | 97 | 100 | 80 | Depression | 1 | 81 | 81 | 84 | 90 | 96 | - |
|  | 2 | 100 | 67 | 100 | 79 | 100 | 79 |  | 2 | 83 | 73 | 89 | 71 | 84 | - |
|  | 3 | 100 | 91 | 100 | 83 | 100 | 88 |  | 3 | 80 | 82 | 93 | 67 | 90 | - |
|  | 4 | 100 | 69 | 100 | 73 | 100 | 73 |  | 4 | 90 | 69 | 90 | 55 | 100 | - |
|  | 5 | 100 | 83 | 100 | 100 | 100 | 60 |  | 5 | 100 | 83 | 100 | 80 | 33 | - |
| Pain location | 1 | 91 | 81 | 97 | 83 | 96 | 76 | Dependence on others | 1 | 81 | 73 | 71 | 86 | - | - |
|  | 2 | 100 | 67 | 95 | 64 | 89 | 79 |  | 2 | 65 | 67 | 53 | 57 | - | - |
|  | 3 | 100 | 86 | 100 | 92 | 100 | 88 |  | 3 | 75 | 91 | 57 | 83 | - | - |
|  | 4 | 80 | 69 | 80 | 73 | 70 | 73 |  | 4 | 60 | 46 | 50 | 45 | - | - |
|  | 5 | 100 | 83 | 100 | 100 | 100 | 100 |  | 5 | 100 | 83 | 67 | 20 | - | - |

|  |  | **Round 1** | | **Round 2** | | **Round 3** | |  |  | **Round 1** | | **Round 2** | | **Round 3** | |
| --- | --- | --- | --- | --- | --- | --- | --- | --- | --- | --- | --- | --- | --- | --- | --- |
| **Outcome**^±^ | **Group*** | 5PT^a^ | 9PT^b^ | 5PT^a^ | 9PT^b^ | 5PT^a^ | 9PT^b^ | **Outcome**^±^ | **Group*** | 5PT^a^ | 9PT^b^ | 5PT^a^ | 9PT^b^ | 5PT^a^ | 9PT^b^ |
| Full pain recovery | 1 | 81 | 73 | 71 | 72 | 64 | 80 | Emotional symptoms | 1 | 74 | 81 | 74 | 90 | 75 | - |
|  | 2 | 61 | 40 | 74 | 50 | 58 | 50 |  | 2 | 83 | 67 | 79 | 64 | 79 | - |
|  | 3 | 100 | 91 | 100 | 83 | 90 | 75 |  | 3 | 80 | 86 | 79 | 83 | 80 | - |
|  | 4 | 60 | 77 | 60 | 73 | 60 | 64 |  | 4 | 90 | 77 | 70 | 55 | 70 | - |
|  | 5 | 80 | 67 | 67 | 80 | 67 | 60 |  | 5 | 60 | 83 | 67 | 40 | 33 | - |
| **Function/ disability/ activity limitation** | 1 | 95 | 90 | 100 | 100 | 100 | 100 | **Fear avoidance** | 1 | 86 | 79 | 90 | 83 | 93 | 88 |
|  | 2 | 100 | 87 | 100 | 100 | 100 | 100 |  | 2 | 87 | 60 | 74 | 57 | 79 | 64 |
|  | 3 | 95 | 91 | 93 | 92 | 90 | 75 |  | 3 | 80 | 86 | 86 | 83 | 90 | 63 |
|  | 4 | 100 | 85 | 100 | 100 | 100 | 100 |  | 4 | 70 | 77 | 80 | 73 | 80 | 82 |
|  | 5 | 100 | 83 | 100 | 100 | 100 | 100 |  | 5 | 100 | 83 | 100 | 80 | 67 | 80 |
| Physical activity levels/ exercise limitations | 1 | 93 | 85 | 97 | 97 | 100 | 100 | Frustration | 1 | 72 | 67 | 35 | 55 | - | - |
|  | 2 | 96 | 80 | 95 | 93 | 84 | 93 |  | 2 | 43 | 40 | 32 | 7 | - | - |
|  | 3 | 90 | 91 | 100 | 92 | 80 | 75 |  | 3 | 80 | 82 | 71 | 67 | - | - |
|  | 4 | 70 | 77 | 70 | 100 | 80 | 100 |  | 4 | 40 | 23 | 40 | 27 | - | - |
|  | 5 | 100 | 83 | 100 | 80 | 100 | 80 |  | 5 | 60 | 50 | 67 | 60 | - | - |
| Need for mobility aid | 1 | 81 | 73 | 84 | 72 | 75 | - | Pain catastrophizing | 1 | 81 | 73 | 84 | 83 | 96 | - |
|  | 2 | 70 | 53 | 74 | 43 | 58 | - |  | 2 | 83 | 80 | 74 | 57 | 74 | - |
|  | 3 | 85 | 73 | 79 | 75 | 60 | - |  | 3 | 80 | 73 | 86 | 67 | 90 | - |
|  | 4 | 80 | 46 | 60 | 64 | 20 | - |  | 4 | 90 | 85 | 80 | 64 | 90 | - |
|  | 5 | 100 | 67 | 67 | 60 | 100 | - |  | 5 | 100 | 83 | 67 | 80 | 67 | - |
| Perceived body imbalance | 1 | 47 | 56 | 26 | 34 | - | - | Self-efficacy | 1 | 84 | 81 | 81 | 86 | 86 | - |
|  | 2 | 48 | 33 | 26 | 29 | - | - |  | 2 | 83 | 73 | 79 | 86 | 68 | - |
|  | 3 | 70 | 68 | 57 | 58 | - | - |  | 3 | 85 | 77 | 79 | 50 | 80 | - |
|  | 4 | 40 | 15 | 0 | 27 | - | - |  | 4 | 70 | 85 | 70 | 73 | 70 | - |
|  | 5 | 100 | 17 | 33 | 40 | - | - |  | 5 | 100 | 83 | 67 | 60 | 67 | - |

|  |  | **Round 1** | | **Round 2** | | **Round 3** | |  |  | **Round 1** | | **Round 2** | | **Round 3** | |
| --- | --- | --- | --- | --- | --- | --- | --- | --- | --- | --- | --- | --- | --- | --- | --- |
| **Outcome**^±^ | **Group*** | 5PT^a^ | 9PT^b^ | 5PT^a^ | 9PT^b^ | 5PT^a^ | 9PT^b^ | **Outcome**^±^ | **Group*** | 5PT^a^ | 9PT^b^ | 5PT^a^ | 9PT^b^ | 5PT^a^ | 9PT^b^ |
| Sexual function | 1 | 86 | 79 | 87 | 79 | 75 | - | Well-being | 1 | 81 | 83 | 74 | 90 | - | - |
|  | 2 | 70 | 67 | 84 | 79 | 68 | - |  | 2 | 65 | 60 | 47 | 57 | - | - |
|  | 3 | 80 | 64 | 79 | 42 | 70 | - |  | 3 | 85 | 86 | 86 | 58 | - | - |
|  | 4 | 80 | 69 | 70 | 73 | 70 | - |  | 4 | 60 | 77 | 60 | 64 | - | - |
|  | 5 | 100 | 83 | 67 | 60 | 100 | - |  | 5 | 100 | 83 | 67 | 80 | - | - |
| **Health related quality of life** | 1 | 95 | 83 | 94 | 97 | 100 | 96 | Fatigue | 1 | 70 | 60 | 65 | 52 | - | - |
|  | 2 | 100 | 87 | 100 | 86 | 100 | 86 |  | 2 | 65 | 67 | 42 | 21 | - | - |
|  | 3 | 85 | 82 | 86 | 83 | 80 | 75 |  | 3 | 90 | 82 | 71 | 75 | - | - |
|  | 4 | 100 | 85 | 100 | 100 | 100 | 100 |  | 4 | 70 | 62 | 60 | 55 | - | - |
|  | 5 | 100 | 83 | 100 | 100 | 100 | 80 |  | 5 | 80 | 67 | 100 | 60 | - | - |
| Health status | 1 | 81 | 79 | 81 | 97 | 79 | 96 | Sleep function | 1 | 86 | 79 | 100 | 90 | 100 | 88 |
|  | 2 | 83 | 80 | 79 | 86 | 84 | 64 |  | 2 | 100 | 80 | 100 | 71 | 100 | 79 |
|  | 3 | 85 | 77 | 93 | 75 | 70 | 50 |  | 3 | 100 | 91 | 100 | 75 | 100 | 63 |
|  | 4 | 90 | 85 | 90 | 91 | 90 | 91 |  | 4 | 90 | 92 | 90 | 82 | 90 | 82 |
|  | 5 | 100 | 83 | 100 | 60 | 67 | 100 |  | 5 | 100 | 83 | 100 | 80 | 100 | 100 |
| Family life impact | 1 | 93 | 79 | 97 | 93 | 93 | 84 | Work ability | 1 | 93 | 83 | 94 | 90 | 96 | 96 |
|  | 2 | 87 | 73 | 79 | 71 | 95 | 50 |  | 2 | 100 | 80 | 89 | 79 | 100 | 86 |
|  | 3 | 90 | 91 | 100 | 83 | 100 | 63 |  | 3 | 100 | 73 | 100 | 83 | 80 | 75 |
|  | 4 | 90 | 85 | 100 | 73 | 100 | 64 |  | 4 | 100 | 92 | 100 | 91 | 100 | 91 |
|  | 5 | 100 | 83 | 100 | 60 | 100 | 80 |  | 5 | 80 | 83 | 100 | 80 | 100 | 100 |
| Social life impact | 1 | 91 | 81 | 87 | 83 | 71 | 76 | Work performance | 1 | 86 | 73 | 84 | 66 | 75 | - |
|  | 2 | 87 | 67 | 84 | 64 | 84 | 50 |  | 2 | 78 | 53 | 79 | 50 | 53 | - |
|  | 3 | 85 | 86 | 86 | 75 | 80 | 50 |  | 3 | 95 | 73 | 86 | 67 | 90 | - |
|  | 4 | 80 | 85 | 90 | 82 | 90 | 55 |  | 4 | 70 | 77 | 70 | 45 | 50 | - |
|  | 5 | 100 | 83 | 67 | 80 | 100 | 100 |  | 5 | 80 | 67 | 67 | 20 | 33 | - |

|  |  | **Round 1** | | **Round 2** | | **Round 3** | |  |  | **Round 1** | | **Round 2** | | **Round 3** | |
| --- | --- | --- | --- | --- | --- | --- | --- | --- | --- | --- | --- | --- | --- | --- | --- |
| **Outcome**^±^ | **Group*** | 5PT^a^ | 9PT^b^ | 5PT^a^ | 9PT^b^ | 5PT^a^ | 9PT^b^ | **Outcome**^±^ | **Group*** | 5PT^a^ | 9PT^b^ | 5PT^a^ | 9PT^b^ | 5PT^a^ | 9PT^b^ |
| Patient satisfaction with life | 1 | 91 | 88 | 94 | 93 | 82 | 84 | Analgesia use | 1 | 72 | 67 | 77 | 52 | - | - |
|  | 2 | 70 | 73 | 47 | 71 | 74 | 86 |  | 2 | 70 | 87 | 63 | 36 | - | - |
|  | 3 | 90 | 91 | 93 | 92 | 70 | 63 |  | 3 | 90 | 64 | 71 | 67 | - | - |
|  | 4 | 50 | 77 | 70 | 73 | 50 | 64 |  | 4 | 60 | 77 | 60 | 45 | - | - |
|  | 5 | 80 | 83 | 100 | 100 | 67 | 80 |  | 5 | 80 | 83 | 67 | 80 | - | - |
| Patient satisfaction with treatment | 1 | 84 | 88 | 84 | 90 | 61 | 88 | Cost | 1 | 70 | 65 | 58 | 55 | - | - |
|  | 2 | 78 | 53 | 74 | 50 | 68 | 50 |  | 2 | 65 | 73 | 53 | 64 | - | - |
|  | 3 | 90 | 91 | 93 | 92 | 90 | 75 |  | 3 | 80 | 77 | 79 | 58 | - | - |
|  | 4 | 60 | 62 | 60 | 64 | 40 | 55 |  | 4 | 70 | 85 | 60 | 64 | - | - |
|  | 5 | 80 | 83 | 67 | 100 | 33 | 80 |  | 5 | 100 | 83 | 100 | 80 | - | - |
| Healthcare utilisation | 1 | 70 | 79 | 77 | 62 | 64 | - | Maternal adverse outcomes/ undesirable effects | 1 | 79 | 63 | 71 | 62 | 68 | - |
|  | 2 | 74 | 73 | 68 | 57 | 68 | - |  | 2 | 74 | 67 | 63 | 43 | 32 | - |
|  | 3 | 95 | 77 | 79 | 75 | 90 | - |  | 3 | 85 | 64 | 71 | 50 | 60 | - |
|  | 4 | 100 | 85 | 80 | 55 | 70 | - |  | 4 | 60 | 62 | 50 | 36 | 40 | - |
|  | 5 | 80 | 67 | 33 | 60 | 33 | - |  | 5 | 80 | 50 | 100 | 60 | 100 | - |
| Anthropomorphic outcomes | 1 | 30 | 23 | 29 | 10 | - | - | Unborn/ born child adverse events/ undesirable effects | 1 | 60 | 60 | 52 | 31 | - | - |
|  | 2 | 65 | 33 | 21 | 14 | - | - |  | 2 | 65 | 53 | 32 | 36 | - | - |
|  | 3 | 35 | 45 | 14 | 33 | - | - |  | 3 | 80 | 50 | 79 | 50 | - | - |
|  | 4 | 50 | 38 | 30 | 9 | - | - |  | 4 | 50 | 38 | 30 | 27 | - | - |
|  | 5 | 80 | 67 | 33 | 100 | - | - |  | 5 | 80 | 50 | 67 | 20 | - | - |
| Body flexibility | 1 | 28 | 44 | 19 | 28 | - | - | Breathing function | 1 | - | - | 48 | 52 | - | - |
|  | 2 | 48 | 33 | 16 | 14 | - | - |  | 2 | - | - | 42 | 21 | - | - |
|  | 3 | 60 | 59 | 14 | 50 | - | - |  | 3 | - | - | 64 | 25 | - | - |
|  | 4 | 40 | 31 | 10 | 0 | - | - |  | 4 | - | - | 0 | 0 | - | - |
|  | 5 | 80 | 17 | 67 | 20 | - | - |  | 5 | - | - | 33 | 60 | - | - |

|  |  | **Round 1** | | **Round 2** | | **Round 3** | |  |  | **Round 1** | | **Round 2** | | **Round 3** | |
| --- | --- | --- | --- | --- | --- | --- | --- | --- | --- | --- | --- | --- | --- | --- | --- |
| **Outcome**^±^ | **Group*** | 5PT^a^ | 9PT^b^ | 5PT^a^ | 9PT^b^ | 5PT^a^ | 9PT^b^ | **Outcome**^±^ | **Group*** | 5PT^a^ | 9PT^b^ | 5PT^a^ | 9PT^b^ | 5PT^a^ | 9PT^b^ |
| Functional mobility | 1 | 79 | 60 | 94 | 55 | - | - | Clinical findings on Motion Palpation/ joint play of Pelvic Girdle joints | 1 | - | - | 68 | 52 | - | - |
|  | 2 | 87 | 60 | 74 | 29 | - | - |  | 2 | - | - | 42 | 36 | - | - |
|  | 3 | 75 | 73 | 64 | 58 | - | - |  | 3 | - | - | 64 | 67 | - | - |
|  | 4 | 70 | 69 | 70 | 36 | - | - |  | 4 | - | - | 20 | 18 | - | - |
|  | 5 | 100 | 50 | 100 | 60 | - | - |  | 5 | - | - | 100 | 60 | - | - |
| Gait endurance | 1 | 86 | 71 | 94 | 69 | 82 | - | Coping strategies/ coping styles | 1 | - | - | 77 | 76 | 68 | - |
|  | 2 | 83 | 53 | 89 | 36 | 74 | - |  | 2 | - | - | 74 | 79 | 53 | - |
|  | 3 | 85 | 77 | 79 | 67 | 90 | - |  | 3 | - | - | 86 | 50 | 80 | - |
|  | 4 | 80 | 46 | 80 | 27 | 70 | - |  | 4 | - | - | 70 | 64 | 60 | - |
|  | 5 | 100 | 67 | 67 | 60 | 100 | - |  | 5 | - | - | 33 | 80 | 33 | - |
| Gait speed | 1 | 67 | 58 | 39 | 41 | - | - | Global perceived improvements/ global rate of change | 1 | - | - | 71 | 86 | 64 | - |
|  | 2 | 52 | 47 | 37 | 29 | - | - |  | 2 | - | - | 89 | 64 | 63 | - |
|  | 3 | 75 | 73 | 57 | 58 | - | - |  | 3 | - | - | 79 | 42 | 80 | - |
|  | 4 | 60 | 46 | 20 | 9 | - | - |  | 4 | - | - | 80 | 45 | 70 | - |
|  | 5 | 60 | 33 | 33 | 20 | - | - |  | 5 | - | - | 67 | 100 | 0 | - |
| New-born outcomes | 1 | 26 | 25 | 19 | 7 | - | - | Goal attainment | 1 | - | - | 81 | 79 | 64 | - |
|  | 2 | 39 | 7 | 26 | 21 | - | - |  | 2 | - | - | 58 | 71 | 58 | - |
|  | 3 | 55 | 45 | 36 | 50 | - | - |  | 3 | - | - | 93 | 50 | 80 | - |
|  | 4 | 20 | 23 | 20 | 0 | - | - |  | 4 | - | - | 40 | 64 | 40 | - |
|  | 5 | 20 | 0 | 33 | 0 | - | - |  | 5 | - | - | 100 | 100 | 67 | - |
| Outcomes from pain provocation /location Tests | 1 | 88 | 63 | 90 | 62 | - | - | Likelihood of planning subsequent pregnancies due to risk of reoccurrence | 1 | - | - | 52 | 59 | - | - |
|  | 2 | 87 | 67 | 79 | 64 | - | - |  | 2 | - | - | 42 | 29 | - | - |
|  | 3 | 80 | 77 | 50 | 58 | - | - |  | 3 | - | - | 79 | 58 | - | - |
|  | 4 | 50 | 62 | 50 | 73 | - | - |  | 4 | - | - | 30 | 36 | - | - |
|  | 5 | 100 | 83 | 100 | 80 | - | - |  | 5 | - | - | 67 | 60 | - | - |

|  |  | **Round 1** | | **Round 2** | | **Round 3** | |  |  | **Round 1** | | **Round 2** | | **Round 3** | |
| --- | --- | --- | --- | --- | --- | --- | --- | --- | --- | --- | --- | --- | --- | --- | --- |
| **Outcome**^±^ | **Group*** | 5PT^a^ | 9PT^b^ | 5PT^a^ | 9PT^b^ | 5PT^a^ | 9PT^b^ | **Outcome**^±^ | **Group*** | 5PT^a^ | 9PT^b^ | 5PT^a^ | 9PT^b^ | 5PT^a^ | 9PT^b^ |
| Posture | 1 | 70 | 63 | 68 | 48 | - | - | Motor control/ movement strategies/ movement patterns | 1 | - | - | 90 | 72 | 82 | - |
|  | 2 | 78 | 47 | 53 | 14 | - | - |  | 2 | - | - | 68 | 50 | 42 | - |
|  | 3 | 90 | 77 | 64 | 42 | - | - |  | 3 | - | - | 93 | 58 | 80 | - |
|  | 4 | 50 | 31 | 30 | 0 | - | - |  | 4 | - | - | 20 | 64 | 30 | - |
|  | 5 | 100 | 17 | 67 | 60 | - | - |  | 5 | - | - | 100 | 80 | 100 | - |
| Pubis Symphysis mobility | 1 | 40 | 31 | 23 | 34 | - | - | Muscle tightness | 1 | - | - | 61 | 69 | - | - |
|  | 2 | 39 | 27 | 32 | 7 | - | - |  | 2 | - | - | 26 | 57 | - | - |
|  | 3 | 80 | 73 | 64 | 42 | - | - |  | 3 | - | - | 86 | 50 | - | - |
|  | 4 | 30 | 38 | 30 | 18 | - | - |  | 4 | - | - | 20 | 27 | - | - |
|  | 5 | 60 | 33 | 0 | 0 | - | - |  | 5 | - | - | 67 | 60 | - | - |
| Maternal pregnancy outcomes | 1 | 56 | 60 | 58 | 38 | - | - | Need for additional supports | 1 | - | - | 48 | 55 | - | - |
|  | 2 | 65 | 33 | 21 | 14 | - | - |  | 2 | - | - | 42 | 29 | - | - |
|  | 3 | 65 | 64 | 64 | 58 | - | - |  | 3 | - | - | 93 | 67 | - | - |
|  | 4 | 40 | 54 | 30 | 9 | - | - |  | 4 | - | - | 20 | 45 | - | - |
|  | 5 | 80 | 67 | 33 | 40 | - | - |  | 5 | - | - | 33 | 40 | - | - |
| Muscle endurance | 1 | 65 | 63 | 61 | 59 | - | - | Outcomes from functional tests | 1 | - | - | 90 | 76 | 82 | - |
|  | 2 | 65 | 40 | 42 | 14 | - | - |  | 2 | - | - | 68 | 71 | 58 | - |
|  | 3 | 70 | 82 | 64 | 67 | - | - |  | 3 | - | - | 93 | 67 | 80 | - |
|  | 4 | 60 | 54 | 30 | 27 | - | - |  | 4 | - | - | 50 | 36 | 20 | - |
|  | 5 | 100 | 33 | 33 | 40 | - | - |  | 5 | - | - | 100 | 40 | 67 | - |
| Muscle strength | 1 | 77 | 60 | 68 | 59 | - | - | Pain duration/ pain pattern | 1 | - | - | 97 | 83 | 96 | 80 |
|  | 2 | 74 | 40 | 47 | 21 | - | - |  | 2 | - | - | 79 | 64 | 84 | 79 |
|  | 3 | 85 | 77 | 71 | 75 | - | - |  | 3 | - | - | 93 | 75 | 100 | 75 |
|  | 4 | 40 | 38 | 20 | 18 | - | - |  | 4 | - | - | 80 | 64 | 80 | 64 |
|  | 5 | 80 | 83 | 67 | 80 | - | - |  | 5 | - | - | 100 | 100 | 100 | 80 |

|  |  | **Round 1** | | **Round 2** | | **Round 3** | |  |  | **Round 1** | | **Round 2** | | **Round 3** | |
| --- | --- | --- | --- | --- | --- | --- | --- | --- | --- | --- | --- | --- | --- | --- | --- |
| **Outcome**^±^ | **Group*** | 5PT^a^ | 9PT^b^ | 5PT^a^ | 9PT^b^ | 5PT^a^ | 9PT^b^ | **Outcome**^±^ | **Group*** | 5PT^a^ | 9PT^b^ | 5PT^a^ | 9PT^b^ | 5PT^a^ | 9PT^b^ |
| Recovery of symptoms | 1 | 91 | 75 | 97 | 83 | 100 | 80 | Patients beliefs about pain/ meaning of complaints to patient | 1 | - | - | 87 | 93 | 71 | - |
|  | 2 | 96 | 60 | 89 | 57 | 89 | 79 |  | 2 | - | - | 63 | 79 | 63 | - |
|  | 3 | 95 | 82 | 100 | 83 | 100 | 88 |  | 3 | - | - | 71 | 67 | 80 | - |
|  | 4 | 90 | 85 | 100 | 73 | 100 | 73 |  | 4 | - | - | 60 | 64 | 40 | - |
|  | 5 | 100 | 67 | 100 | 80 | 67 | 80 |  | 5 | - | - | 100 | 80 | 67 | - |
| Step length | 1 | 44 | 50 | 16 | 31 | - | - | Patient understanding/ knowledge of PGP | 1 | - | - | 84 | 90 | 79 | - |
|  | 2 | 48 | 27 | 5 | 14 | - | - |  | 2 | - | - | 74 | 71 | 63 | - |
|  | 3 | 55 | 68 | 29 | 50 | - | - |  | 3 | - | - | 86 | 67 | 70 | - |
|  | 4 | 30 | 23 | 30 | 9 | - | - |  | 4 | - | - | 60 | 55 | 50 | - |
|  | 5 | 60 | 17 | 33 | 20 | - | - |  | 5 | - | - | 67 | 100 | 67 | - |
| Surgical outcomes | 1 | 37 | 31 | 16 | 14 | - | - | Postural observation | 1 | - | - | 71 | 69 | - | - |
|  | 2 | 35 | 27 | 21 | 14 | - | - |  | 2 | - | - | 47 | 36 | - | - |
|  | 3 | 40 | 59 | 29 | 25 | - | - |  | 3 | - | - | 86 | 67 | - | - |
|  | 4 | 30 | 31 | 10 | 0 | - | - |  | 4 | - | - | 20 | 18 | - | - |
|  | 5 | 80 | 17 | 33 | 60 | - | - |  | 5 | - | - | 67 | 60 | - | - |
| Urinary incontinence | 1 | 84 | 75 | 90 | 83 | 79 | - | Symptoms during menstruation | 1 | - | - | 58 | 55 | - | - |
|  | 2 | 83 | 60 | 79 | 50 | 79 | - |  | 2 | - | - | 26 | 14 | - | - |
|  | 3 | 85 | 77 | 86 | 75 | 80 | - |  | 3 | - | - | 79 | 67 | - | - |
|  | 4 | 70 | 77 | 70 | 64 | 70 | - |  | 4 | - | - | 0 | 9 | - | - |
|  | 5 | 80 | 67 | 100 | 60 | 100 | - |  | 5 | - | - | 67 | 40 | - | - |

S2 Table details the % of stakeholder group that rated an outcome as “important” (4+ on the 5-point Likert scale survey or 7+ on the 9-point Likert scale survey) above in each Delphi round. ^±^ Outcome in final PGP-COS
* Group 1 = clinician; 2 = clinician/researcher; 3 = patient; 4= researcher; 5 = Service provider/policy maker
^a^  5PT = participants responded to Delphi surveys using a 5-point Likert Scale
^b^ 9PT = participants responded to Delphi surveys using a 9-point Likert Scale
