# Supplementary material for: A core outcome set for research and clinical practice in women with pelvic girdle pain: PGP-COS
Source: PLoS One. 2021 Feb 25;16(2):e0247466. doi: 10.1371/journal.pone.0247466 (PMC7906405; doi:10.1371/journal.pone.0247466)
Supplement: Attachment — Submitted filename: Response to Reviewers.docx [file pone.0247466.s003.docx]

Dear Editor and Reviewers,

Thank you for your helpful comments on our revised paper. We have attended to the comments with point by point responses as below, and in further revisions to the manuscript. We hope these meet the requirements for progressing our paper to publication.

­­­­­­­­­­­­­­­_________________________________________________________________________________________

Journal Requirements

1. Please ensure that your manuscript meets PLOS ONE's style requirements, including those for file naming. The PLOS ONE style templates can be found at

<https://journals.plos.org/plosone/s/file?id=wjVg/PLOSOne_formatting_sample_main_body.pdf> and

<https://journals.plos.org/plosone/s/file?id=ba62/PLOSOne_formatting_sample_title_authors_affiliations.pdf>

 Thank you. We have updated formatting to meet PLOS ONE’s style requirements.

2. Please include in your financial disclosure statement the name of the funders of this study (as well as grant numbers if available). At present, this information is only available in the funding section of your manuscript.

Thank you for stating the following in the Funding Section of your manuscript: [We have received funding from the Belgian Chiropractic Union Research Fund ([www.chiropraxie.org](http://www.chiropraxie.org/)) and from the European Centre for Chiropractic Research Excellence ([nikkb.dk/eccre](http://nikkb.dk/eccre)). The funders had no role in the study design, data collection, management, data interpretation, report writing and decision to submit for publication.]

We note that you have provided funding information that is not currently declared in your Funding Statement. However, funding information should not appear in the Acknowledgments section or other areas of your manuscript. We will only publish funding information present in the Funding Statement section of the online submission form. Please remove any funding-related text from the manuscript and let us know how you would like to update your Funding Statement. Currently, your Funding Statement reads as follows: [The funders had no role in study design, data collection and analysis, decision to publish, or preparation of the manuscript.]

We have removed all funding-related text from the manuscript. We would like to change our Funding Statement to read as follows:

[We have received funding from the Belgian Chiropractic Union Research Fund (www.chiropraxie.org) and from the European Centre for Chiropractic Research Excellence (nikkb.dk/eccre). The funders had no role in the study design, data collection, management, data interpretation, report writing and decision to submit for publication.]

3. Please include captions for your Supporting Information files at the end of your manuscript, and update any in-text citations to match accordingly. Please see our Supporting Information guidelines for more information: <http://journals.plos.org/plosone/s/supporting-information>.

Supporting Information files have been added to the end of the manuscript and we have renamed the supporting information file names to match the journal requirements.

________________________________________________________________________________________

Thank you for your thorough summary and complementary words about our manuscript. We have addressed your comments as below.

Reviewer #1: The paper presents results of a Delphi study to establish a Core Outcome Set (COS) for pelvic girdle pain (PGP) in pregnancy and postpartum. There is a lack of standardization in studies on PGP, especially related to the outcome measures selected for use with the pregnant and postpartum population with PGP. This lack of standardization results in disparate results across studies on the population of interest, and consequently, impedes the synthesis and broader analysis of research on PGP in pregnant and postpartum women. This paper has performed a 3-round Delphi study with an in-person consensus meeting involving multiple stakeholders across several countries. Five outcomes were selected by the Delphi group as the proposed PGP-COS: pain frequency, pain intensity/severity, function/disability/activity limitation, health-related quality of life and fear avoidance.

Thank you for a concise summary of our manuscript.

The rationale, methodology, results and implications of the study are presented clearly and logically. The five outcomes selected for the PGP-COS are logical, clinically relevant, feasible and not duplicative. While the constructs for each outcome are related, the overlap is not excessive and is, in fact, complementary when considering the client with PGP as a whole. All refer to the “life impact” domain, rather than outcomes that focus on specific physical impairments, such as muscle strength, flexibility or joint symmetry. This supports the clinical relevance and feasibility of moving forward with the proposed PGP-COS in clinical practice and research. Inclusion of, and further research on, the proposed PGP-COS from an implementation standpoint would add to the current literature on PGP, as the “life impact” of PGP on childbearing women (on a national or global) level is limited.

Thank you for your comments. We agree that implementation and uptake of the proposed PGP-COS will add to the current literature on PGP as the “life impact” of PGP on childbearing women is limited.

One of the biggest challenges in PGP research is the construct of patients’ own expectations of pain during pregnancy and their perceptions of “normal” recovery during the postpartum. While the authors described the online survey process of the first round of the Delphi study, several elements that speak to the constructs of pain, specifically PGP, women’s own expectations, and the cultural aspects of both pain and what is considered “normal” expectations of musculoskeletal pain and functional status in pregnancy and postpartum across countries were not clearly described. This is the biggest challenge to address in this manuscript, as it appears that assumptions may have been made about stakeholders’ shared understanding of PGP and the impact of cultural differences. While the ranking process addressed stakeholders’ shared value of importance for each outcome, it does not address cultural differences or stakeholders’ expectations. These differences or discrepancies in stakeholders’ shared understanding of PGP, expectations and cultural differences may contribute biases to the results of the Delphi study.

Thank you for your comment. We acknowledge that there are country and cultural differences regarding expectations of pain during pregnancy and perceptions of “normal” recovery during the postpartum and note that there are individual differences as well. For example, two patients can have different expectations about recovery from pregnancy related PGP. However, in this phase of our overall study, the objective focus was on ‘what to measure’ rather than ‘how’ to measure, or on variations as a result of country or cultural differences. These aspects may emerge in phase 4 of the project where the focus is on ‘how’ to measure the COS outcomes, and in the context of future individual trials/studies where this COS (hopefully) will be used. We are thus confident, while accepting the important point you raise, that this does not bias the Delphi results, but will have a role in the application of the PGP-COS; a point we will attend to in reporting phase 4 of the project (‘how’ to measure the outcomes in the COS).

The presence and targeted inclusion of international stakeholders is a strength of this Delphi study. The authors have addressed study limitations of using English only survey tools and having fewer participants than projected involved the final in-person consensus meeting. Additional sources of potential bias and limitations in the generalizability of the proposed PGP-COS, as a result, should be considered. Participants were predominantly: clinicians and clinical researchers, female, middle age, and from Ireland, Sweden, USA and Canada. Information on countries’ maternal health systems, specifically public and health provider knowledge of PGP, as well as reimbursement and access to physiotherapy services for PGP in pregnancy and postpartum, may identify important characteristics of stakeholder countries who responded to the Delphi survey compared to those who did not. A narrative of the authors’ perspectives on the characteristics of participants and how those characteristics are representative of the larger community of interest would add to the manuscript.

Thank you for raising this point. We agree that only language and fewer participants than expected in the consensus meeting are not the only limitations in larger uptake from different nations. Additionally, partial reasons for less uptake in some countries can also be attributed to less awareness about the condition both at individual and healthcare system levels. This is reflective of provider knowledge and access to services. We have addressed this as a further limitation to our study in our manuscript.

The link proposed between development of the PGP-COS and evaluating “preventative” strategies for PGP during pregnancy and postpartum is unclear. The PGP-COS will measure “life impact” of PGP, in patients who have already or are currently experiencing PGP in pregnancy or postpartum. Additional description of the link between use of the PGP-COS and preventative strategies is needed.

Thank you. For clarity we have removed references to “preventative strategies” and refer now to a COS for interventions for PGP. We have slightly tweaked the ‘Aim’ also to clarify this further, which now reads; The aim of our study was to develop a consensus-based COS for PGP during pregnancy and postpartum that should be used, as a minimum, for research and clinical practice.

Reviewer #2: This manuscript describes a diligently methodical process of identifying a well-vetted core outcome set for prenatal and postpartum pelvic girdle pain. I found the manuscript to be well written and to describe a clinically sound process.

One improvement that could be made would be references in the introduction to specific ways in which lack of a COS for PGP has impacted the literature on this problem. For those of us who do not regularly evaluate or manage PGP, this might be helpful.

Thank you for your time in reviewing our manuscript. We have added specific comments to the introduction (paragraph 1) in which the lack of a PGP COS has impacted the literature for clarity to those who do not regularly evaluate or manage PGP.
