# Supplementary material for: A core outcome set for research and clinical practice in women with pelvic girdle pain: PGP-COS
Source: PLoS One. 2021 Feb 25;16(2):e0247466. doi: 10.1371/journal.pone.0247466 (PMC7906405; doi:10.1371/journal.pone.0247466)
Supplement: Attachment — Submitted filename: Response to Reviewers.docx [file pone.0247466.s004.docx]

Dear Editor and Reviewers,

Thank you for your helpful comments on our revised paper. We have attended to the comments with point by point responses as below, and in further revisions to the manuscript. We hope these meet the requirements for progressing our paper to publication.

Editor:

Thank you for addressing the reviewers' comments.   Please see and consider further reviewer suggestions below, in particular,  including "comparison of the round 3 stakeholders' response, or reporting round 3 results and presenting how they compare to the final 11 participants' consensus opinions", to address the issue discussed.

Thank you. We have addressed this comment of reviewer 1 below. While direct statistical comparison of round 3 responses with the results of the consensus meeting was not conducted since it was not feasible nor appropriate (see reasons in response to reviewer’s comment), readers can easily compare the round 3 and consensus meeting results from the information provided in table 4 and supplementary table 2. This is now also mentioned in the manuscript.

Reviewer #1: The authors have addressed prior feedback early on in the paper well. Minor edits are suggested below.

Inconsistencies noted in the organization of preliminary outcomes (Table 1), with some lack of clarity on the transition between rounds.

1. The "Pathophysiological Manifestations" title in column 3 may also include aspects of pain, depression and anxiety, sleep disturbance, emotional symptoms, confidence and wellbeing, etc. These outcomes are already listed in "Life Impact". There appears to be some redundancy across those 2 columns, and in categorizing outcomes, the authors might consider including "Pathophysiological Manifestations" into "Life Impact" and leave the clinical tests in this freestanding category.

Thank you for your comment. Our outcome categorisation and subsequent table headings adhere to the OMERACT 2.0 Filter Framework (Boers et al. 2014). In this framework Life Impact and Pathophysiological Manifestations are established as separate domains. According to this framework, aspects of pain, depression and anxiety, sleep disturbance, emotional symptoms, confidence and wellbeing, etc. are classified as Life Impact domain outcomes (Fig 1; Boers et al 2014). We have kept Table 1 column titles as is for consistency with this framework and also the previous publication in which possible outcomes for the PGP-COS were identified (Wuytack and O’Donovan. 2019).

2. The outcomes included in the third column - "Pathophysiological Manifestations/Clinical Tests" of maternal pregnancy outcome, newborn outcome and surgical outcome do not seem to be appropriately categorized. Perhaps these should move to "Adverse Events and/or Medical Outcomes" to capture these factors, or be assigned a stand-alone column for Medical Outcomes.

Thank you for your comment. As described above, the outcomes are categorised according to the OMERACT 2.0 filter framework (Boers et al. 2014) and our previous publication identifying potential outcomes for the Delphi study (Wuytack and O’Donovan. 2019). We have kept categorisation as is for consistency as well as the outcomes were grouped as such during the Delphi Study. We have included a statement addressing this in the main text (page 6).

3. The "anthropometric outcome" is unclear - does this refer to weight gain and/or BMI during pregnancy?

Thank you for your comment. Anthropometric outcomes were identified in our previous systematic review (Wuytack and O’Donovan. 2019) as well as in Supplementary Table 1 of this manuscript. Anthropometric outcomes were defined as measurements of size and proportion of the human body for example, height, weight, BMI, etc.. Reference to S1 Table is made in the Delphi Procedure section of methods. It is not referenced in Table 1 as this is just a list of the potential outcomes.

4. "Recovery of symptoms" seems too broad. This may then include "full pain recovery" which is listed in "Life Impact". These categories should be mutually exclusive, meaning that outcomes listed across may be related, but cannot be interchanged for each other. Perhaps some recoding of categories and/or re-evaluation of outcomes is needed.
Thank you comment. Full pain recovery in our Delphi survey was defined as “being pain free/no longer in pain after treatment.” This is separate to recovery of symptoms as symptoms can include more than pain. The definition is also included in Supplementary Table 1. As mentioned above we have kept categorisation as is for consistency across publications as well as this is how outcomes were identified in studies included the systematic review leading into the Delphi (Wuytack & O’Donovan 2019) and were presented to participants during the Delphi Study.

Rating scales used to assess preliminary outcomes in Round 1 and 2 of the Delphi study include both a 5-point and a 9-point scale, administered randomly. Explanation of the rationale for use of the 2 different scales is suggested. Although it is mentioned this will be presented in a separate paper, it is a point of confusion for this study's methodology.

Thank you for your comment. We have added an explanation of our rational for using the two scales in the main text. The findings from the embedded methodological study have since been published and we have addressed the text appropriately for reader’s reference (page 9).

The limitations of the consensus meeting are again, striking. To base the final round of inclusion on the opinions of 11 individuals, when 132 stakeholders completed round 3, seems potentially biased. This final consensus round, arguably the most important and most impactful round on results of this Delphi study, represented the opinions of only 17% of the stakeholders. While this limitation is explained because of travel emergencies, it is suggested the authors perform additional statistical analyses on the results of round 3 and/or explore a remote voting process for the 83% of round 3 stakeholders whose voices and opinions were not heard in this final round of the Delphi process. Perhaps a comparison of the round 3 stakeholders' response, or a virtual consensus meeting may be needed, or reporting round 3 results and presenting how they compare to the final 11 participants' consensus opinions.

Thank you for your comment. We understand your concern regarding 11 participants in our face-to-face consensus meeting. However, we adhered to the current methodological guidelines for Core Outcome Set development developed by COMET (Core Outcome Measures in Effectiveness Trials Initiative). According to these guidelines, the face-to-face consensus meeting includes a subset of participants. These guidelines do not include a required number of participants. However, Waggoner, Carline and Durning. 2016 have previously explored the optimal consensus panel size and have determined 5-11 members is recommended. We had referenced this in our limitations but since realised that the article was incorrectly referenced. This has been rectified and is now included in our reference list. In line with these recommendations, most other previous COS development projects use a similar size sub-group of Delphi participants for the face-to-face. It is also important to note that while the COMET handbook does recommend a face-to-face meeting, they also refer to a review that identified that not all COS include this step in their development (page 24 of the handbook). While the face-to-face meeting may seem to be the most important and most impactful round, the three previous rounds of the Delphi study are just as important. The Delphi process is a consensus process itself.

In our Supplemental Table 2 (S2 Table) we have provided how all stakeholder groups voted throughout all rounds and have also highlighted the final five outcomes included in the PGP-COS. From this table, comparisons between the consensus meeting and round 3 responses can be made. However, *statistical* comparison of round 3 responses with the results of the consensus meeting was not feasible nor appropriate due to the following reasons: 1) This is not recommended in current COS development methodology (COMET handbook); 2) The consensus definition in the Delphi and face-to-face meeting were different in that for the Delphi >70% of only 3 stakeholder groups (including at least the patient group) needed to rate an outcome as important, while for the meeting >70% of all participants had to vote an outcome as important (This is line with current COS development guidelines; 3) The 2 different scales we used makes for the embedded methodological study. For example 6 outcomes that were voted on in the consensus meeting were only voted on by one of the scale groups (not both).

Some outcomes that were rated high by all stakeholder groups in Round 3 were not subsequently included in the final COS during the face-to-face consensus meeting. However, this is expected during the consensus process aiming at identifying a small number of core outcomes that should and could be measured in all trials and in clinical practice. As more and more rounds are incorporated during the whole consensus process, more and more outcomes begin to not reach consensus. This is evidenced by starting with 53 outcomes in Round 1 to only 20 outcomes reaching consensus in round 3. It is therefore expected that the number of outcomes reaching consensus in round 3 would reduce during the final face-to-face meeting. Several outcomes from round 3 (e.g. sexual functioning) were also not included in the final COS because it was agreed by the participants that they formed part of other outcomes that were included in the final COS (e.g. activity limitations) (this is mentioned in the discussion on page 16). Finally, it is also important to note that only 13% of Delphi participants expressed interest in participating in the face-to-face consensus meeting when asked at completion of the survey (being informed that travel and accomodation costs would be funded). As such, it would not be feasible or methodologically appropriate to include all participants from round 3 in the face-to-face consensus meeting.

Additionally, of the 11 participants at the consensus meeting, 6 (54.5%) were researchers. This also contributes to bias. While the authors disclose participants at the consensus meeting holding multiple roles, this point should be made explicit.

Thank you for your comment. Table 5 reports that 3 participants were researchers, 3 participants were clinicians and 4 participants were clinician/researchers. While having a larger number of researchers may contribute to bias, there is currently no consensus on who should be involved in the face-to-face meeting (COMET Handbook). Table 5 also reports the multiple roles by the 11 participants (including participants who were patients in addition to belonging to other stakeholder group(s)). We have addressed the text to be more explicit about the multiple roles of these participants (page 14 and 18).
